# Hospital and Institutional News

**Published:** 1919-03-29

**Authors:** 


### HOSPITAL AND INSTITUTIONAL NEWS.

### MEDICAL ASSOCIATION MEETING.

The programme of the Clinical Meeting of the British Medical Association to be held in London from April 8 to 11 has now appeared and the sectional meetings are to take place at the Royal College of Science, South Kensington. Among the features most interesting to the practitioner which will occur during this time are included a popular lecture by General Cuthbert Wallace on the Surgery of War, a discussion on War Neuroses on Wednesday, April 9, 10 a.m. to 1 p.m.; a full review of the influenza situation by Sir Wilmot Herringham, Captain Greenwood, Major Bowman, and others on April 10, 11 a.m. to 1 p.m. Sir William Osler, on the following day from 10 a.m. to 11.30 a.m., will preside over a discussion on venereal disease control, and immediately after Sir James Mackenzie will take the chair at a meeting in connection with cardio vascular disease. On the surgical side, April 9 is set down for Gunshot Wounds of the Chest, on the next day shock will be dealt with, Professor Bayliss speaking, and on April 11, Sir Robert Jones will preside over a review of Orthopædic Surgery. Preventive medicine will be dealt with in connection with influenza, the dysenteries, malaria, etc., and on Friday at 12 noon, in this section, a communication will be made upon the "filter-passing" virus in influenza and other diseases. Arrangements are complete for many demonstrations and exhibitions, and we heartily congratulate the Association upon its excellent programme.

### THE FELLOWSHIP OF MEDICINE.

The recently-formed Inter-Allied Fellowship of Medicine has already achieved many creditable things, and not the least important are the arrangements whereby medical men in America are being brought into closer touch with our own. Functions held last week tended to further the good fellowship and bring before every one the essential requirements of the times. In a scientific profession tending every day to extend its researches over wider fields and to probe more deeply into the mysteries of human existence, the only way in which efficiency can be attained is by the removal of every barrier to the free interchange of thoughts and opinions originating the world over. It is not only the broader view which is needed, but also the wider, more easily accessible general knowledge. If the two great Anglo-Saxon Allies commence, with true sympathy, to work on a basis of goodwill and perfect understanding, it will be not only a great advance in the comradeship we wish to develop but of inestimable value to the advance of science. On all hands there are signs that our most sanguine hopes will be fulfilled, for leading men on both sides of the Atlantic are practically unanimous in their support. International post-graduate study seems to hold one at least of the keys to the position, and arrangements are in process of completion whereby this may become not only a possibility for the medical man but also the usual custom.

### A GLUT OF STUDENTS AT CARDIFF.

The measure of success which hospital work in Cardiff has lately attained has been the measure of the goodwill opposed to the difficulties confronting it. Therefore every difficulty is rather an earnest of further success than of another obstacle. The number of medical students at University College, Cardiff, has risen since the armistice to 160. It was only eighty before the war. The women have crowded in. But there is a ludicrous absence of accommodation. The crush is so great that classes probably will have to be repeated twice a day. It is—we are speaking of the novelty—the "monstrous" regiment of women which is bringing matters to a head. Architecturally speaking, only the physiological block of the Welsh National School of Medicine, which is close to King Edward VII. Hospital, Cardiff, has yet been built. It will hardly be ready for students before the end of six months; and the rest of the building hardly for another couple of years. The Dean of the Medical School, Colonel D. Hepburn, C.M.G., takes a grave view, as well he may. But difficulties have been the school's best friends hitherto, and we hope that, with such men as Colonel Hepburn and Colonel Bruce Vaughan, they will again prove their value.

### A DIAGNOSTIC HOSPITAL.

In New York there is shortly to open a hospital specialised for the rapid diagnosis of disease and capable of handling some five hundred cases daily. This institution is the first of its kind in America, and we await the details of its organisation and working efficiency with considerable interest. With the recent extension of the list of notifiable diseases, the now frequent appearance of relatively rare kinds of infection and the general influx of war sickness to this country, the possible value of such hospitals to Great Britain is obvious. The much-hoped-for developments in preventive medicine could be helped in many ways by such specialised and rapid work, while the advantage to the panel scheme from both doctors' and patients' point of view might be considerable. One would be glad to hear the opinion of authorities in these branches of homeland work upon the possible utility of what appears to us a most valuable institution.

### THE CLEANSING OF PERSONS ACT.

The urgency of compulsory extension of this Act is being brought to notice by a report circulated to members of the House of Commons and arising from investigation by a special sub-committee of the Parliamentary Advisory Council. It is suggested that one of the chief but remediable of housing evils lies in the verminous condition of many homes, not only undermining the health, mind, and education of the people, but also taking an active part in the transmission of disease. Two main lines of reform are demanded, namely, the provision of public cleansing stations by local authorities and the removal of inefficient co-ordination between the different State services into whose province these matters fall. Public institutions can deal temporarily with both adults and children they may have under their care, and legislation gives power to health and school authorities to enforce a satisfactory personal cleanliness for the younger generation, but through neither channel is touched in actual practice the root of the whole matter, the home. Of the menace of disease in connection with demobilisation we have been of late too frequently warned to miss the urgency of these matters. Not only should this Act be made compulsory, and cleansing stations developed in proportion to the requirements of any given area, but also, among many other points, the Town Planning Act of 1909 should be so strengthened that empty verminous houses can be declared unfit for occupation, unclean persons entering institutions and children found in unsatisfactory condition should be reported to authorities who have power compulsorily to investigate and cleanse the homes, and no Housing Bill should be passed which does not provide facilities for dealing with the dirt problem. In almost every quarter existing arrangements are unsatisfactory in the extreme, and we trust that due consideration will be given to this matter.

### THE PREVENTION OF SCURVY AND BERI-BERI.

Evolved during the dietetic researches at the Lister Institute at first, upon the distribution of foodstuffs to the expeditionary forces in desert countries, and later applied to the feeding of children among the civil population, there has been brought forward some interesting evidence as to the distribution of the essential food factors or "vitamines." In the case of beri-beri it was found that these anti-substances were markedly present in seeds of plants and eggs of animals, making provision for the offspring's need during early existence, and that desiccation of the eggs but slightly lowered their value. Generally speaking, the more highly cellular the nature of the food the stronger was the preventive action, meat (muscle), milk, cheese, etc., being relatively low in the scale. In guinea-pigs and monkeys acute scurvy was produced by a diet of cereals with water or sterilised milk. The accepted fact that fresh fruit juices and fresh green vegetables were the richest in the anti-scorbutic element was well borne out. Germination of pulses, substances normally deficient, was found to produce a considerable amount of vitamine and after transportation in the drystate, if these products were allowed to germinate there was no deterioration in food value, and great increase in anti-scorbutic power. Lemons were shown more valuable than limes and, from both the home and service points of view, the great value of the onion, a powerful anti-scorbutic, was fully demonstrated. In the case of children of the poorer classes, use may be made of raw swede juice, the best of many tried, and should be added to the diet of any infants reared on heated or dried milk-products or other vitamine-deficient diet. The researches cover to a large extent well-traversed fields, and the newly-termed "vitamines" have, in principle, long been known, but these contributions are most valuable in details of application and are well worthy of consideration by those interested in welfare problems.

### THE REAL WAY TO REMOVE THE PAUPER TAINT.

Now that the new workhouse infirmary, which was completed at a cost of £8,000 just before the war, has reverted to the Ruthin Board of Guardians, the advice of Mr. H. R. Williams, the Local Government Board's Inspector for Wales, is worth recording. Asked to advise how best to use it, Mr. Williams told the Guardians to clean and paint the institution and then to try to induce the sick poor of the Union to make use of it. In answer to the objection of Mr. Henry Hughes, the chairman even recalled the unwillingness of eligible patients to incur the "taint of pauperism." Mr. Williams pointed out that the law as it stood at present had destroyed all taint of pauperism. The sick of the union, he said, whether poor or not, should avail themselves of the infirmary. If they could pay, much or little, all the better for every one. No decent man or woman need be ashamed to enter it. Besides, those who could keep themselves only when well became in practice (technically) destitute on falling ill, because (we suppose Mr. Williams referred to the shortage of beds) they could not get institutional treatment in the Manchester and Liverpool infirmaries. Mr. Williams said that he was informed that Dr. J. Medwyn Hughes, the medical officer of the Union Infirmary, had no objection to the patients being attended by their own doctors. Elsewhere the jealousies of medical men had, he said, led to a refusal to allow union patients to be healed by any one except the union medical officer. But the way was clear in the present case for the Guardians and Dr. Hughes to come to an arrangement. We hope it will be made. The pauper taint can be best removed not by legislation but by the common sense of all concerned.

### THE NEW JERUSALEM EYE HOSPITAL.

Our recent references to the needs of Jerusalem Eye Hospital make it pleasant to record its reopening by General Allenby, news of which has at last reached England, after much delay. The *Times* notes the uncivil use to which the hospital was put by the Turks; its destruction when they evacuated the city; its prompt rebuilding by the Order of St. John. The opening ceremony was worthy of the occasion. Delegates of the American Red Cross and the Zionist Medical Unit were present. A telegram from the Grand Prior, the Duke of Connaught, was read, and among others Colonel Garner, Director-General of the Egyptian Public Health Department, was decorated with the ribbon of a Knight of Grace. A special visit was paid by General Allenby to the chapel of St. John Underground, once the ancient crypt of Justinian's time, over which the later Greek Church of St. John is at present built. Egypt and ophthalmology are names congruous in sound; and in Palestine and the East the treatment of diseases of the eye is perhaps the most useful work which a hospital can undertake. May the newly built institution maintain and extend the reputation of the old, and escape the interruption of another Armageddon.

### THE GROWTH OF MUNICIPAL HOSPITALS.

The chief flaw in the voluntary system has been the shortage of beds, with its consequent scandal of reducing to medical destitution many deserving cases whose names appear on waiting-lists, not in the records of the wards. The Public Health Acts have long given to municipal bodies the power to remedy this defect, and the voluntary hospital has no excuse for missing the chance which municipal inertia has so long provided. This chance was the *raison d'être* of the voluntary hospital. But the world will wait no longer. Let us, then, with a sympathy which is not unmingled with shame, note what is being done to supply our own deficiency. The proposal of the Health Committee of South Shields provides a typical case. On the recommendation of Dr. Mathieson, M.O.H., plans have been approved to provide at Cleadon Park 100 beds for tuberculous patients, two ward blocks, with a cubicle pavilion and a discharge block for infectious cases. The nurses' quarters and administrative offices will be provided in existing rooms. The present isolation hospitals at the Deans will become, under the scheme, an out-patient centre for maternity, child welfare, and venereal diseases. Later blocks are also planned. It is a big opportunity and an interesting scheme; would that the voluntary system had not missed the credit of it.

### PATIENTS' FEES IN WOMEN'S HOSPITALS.

One of the few hospitals at present which possesses a balance in hand is the Derbyshire Hospital for Women, which has £374 to its credit. An analysis of the accounts shows that an important source of income is the patients' fees, which amounted last year to a grand total of £770. It would be interesting to have further details of the system adopted. A voluntary hospital is a hospital which offers free treatment to the poor, and treatment according to their means to the middle classes. It is, or should be, the resort of all. Its guiding principle is uncommercial. Fee is perhaps an unfortunate term, for the sum paid is, in principle, not part of a bargain, but a thankoffering, according to a patient's resources. Where a hospital depends upon the subscriptions of working men, the latter are apt to degrade themselves by alleging "rights" in return for "payments." We do not want the middle classes to make the same mistake—to claim as a commercial right the treatment which is offered to them as a matter of human decency. It is, therefore, as important for a hospital not to aim at a profit (or even to seem to make one) as it is for the patients, in return for their treatment, to regard it as a complimentary point of honour to be as generous as they can.

### A TEST FOR NURSING HOMES.

The value of a pay hospital can generally be gauged by its prime object: that of an ordinary nursing home is purely commercial, and it is doubtful if modern hospital treatment and skilled nursing can be given on purely commercial lines. If the prime end in view is as high as possible a dividend on the share capital, other considerations, namely those which affect the patient and the nurses, will always be made to take a second place. Thus many so-called nursing homes are merely examples of the most speculative and expensive kind of ruinously bad hotel. If the institution deserves the name of hospital, either it will have an endowment, or the size of the dividend to be sought will be limited by the articles of association to a modest three or four per cent.; patients will bring their own doctors; and doctors will only be allowed to take shares in the concern on carefully drawn conditions. Probably Fitzroy House, Fitzroy Square, London, is still the model by reference to which the status of other pay hospitals and "nursing homes" can be measured.

### ARMY HOSPITALS' ACCOUNTS.

Last year as an experiment three experienced hospital secretaries were commissioned to duplicate the accounts of three large Army hospitals on voluntary hospital lines. We understand that the system is now to be applied to the whole of the British Army. Captain J. W. Pearce, General Superintendent and Secretary of the Birmingham and Midland Eye Hospital, who was stationed at the Cambridge Hospital, Aldershot, has been promoted to the rank of Major, and is now supervising the introduction of the system to the hospitals situated in the Western Command. Captain H. R. S. Dence (Secretary of the Central London Ophthalmic Hospital) remains in charge of the accounts at the Royal Herbert Hospital, Woolwich, and Captain George Watts, of the City of London Hospital for Diseases of the Chest, who worked at the Horton War Hospital, Epsom, resigned his honorary commission at the end of January, after having started the accounts on the new lines. The change in system, although it comes so late in the day, is of much interest to voluntary hospital workers.

### THE LEGACY OF MILITARY CONTROL.

To have an institution evacuated by the military is one thing, to regain possession is quite another, as the Lincoln Board of Guardians has disagreeably found. The delay is expensive and irritating, because it means that old people and children have still to be maintained at the ratepayers' expense in Leicester and Nottingham institutions. The delay has been caused because a Government surveyor was not promptly sent to assess with the local surveyor the financial equivalent of dilapidations. The military have turned Nelson's eye to the Guardians' letters, and Mr. G. Harley has publicly stated his belief that, when the Guardians received the report of their architect and members after their visit, they would find a condition of things for which, if their own officials had been responsible, an inquiry by the Local Government Board would probably be made. It was unanimously agreed to apprise the Local Government Board of the facts, and to warn them of the expense incurred through the indifference (to use no stronger word) of the military.

### HOSPITAL SECRETARY AS COUNTY COUNCILLOR.

In a three-cornered contest in Pontnewynydd Southern Ward, Mr. Tom Morgan, Secretary of the Pontypool and District Hospital, has been elected a Labour representative on the Monmouthshire County Council. Mr. F. A. Smith, J.P., a vice-president, of the hospital, who, with Mrs. Smith, recently gave £500 to the institution, was defeated at the same election after having represented Abersychan Central Ward for many years. Another vice-president at the hospital, Mr. James Winstone, J.P., the Welsh miners' leader, was re-elected.

### PUBLIC-SPIRITED WELSH TRADERS.

A gratifying example of public spirit and generosity on the part of traders was witnessed last week when a deputation attended at the Prince of Wales' Hospital, Cardiff, to present a sum of £100 raised by the Gilfach Goch Chamber of Trade to maintain a bed at the Hospital for two years. Gilfach Goch is a small mining town about twenty miles from Cardiff, and gratification was expressed at the broad-minded generosity of the Chamber of Trade in helping the hospital, which has assisted so many limbless soldiers in Wales and Monmouthshire.

### TWO HOSPITAL SCHEMES IN WALES.

A gratifying announcement was made at the annual meeting of Merthyr General Hospital by Mr. Wm. Griffiths, chairman of the executive board. The problem of accommodation has been very acute at the hospital, and Mr. Griffiths stated that, in view of the fact that the workmen of the borough had, by contributing £2,550 last year, fulfilled their promise of increased support, the board had now in consideration the enlargement of the building. On condition that £3,000 was raised locally in order to form the nucleus of an endowment fund, Messrs. Courtaulds, of Flint, a short while ago offered the town a fine modern house on their estate for use as a cottage hospital. The hospital committee has now been able to claim the gift, a sum of £3,100 having been obtained, while it is anticipated that there will also be forthcoming the £500 required for furnishing and equipping the institution.

### TRANSFER OF WELSH NATIONAL HOSPITAL.

The future of the Welsh National Hospital, Netley, which has been for some time uncertain, is now settled, arrangements having been made for its transfer to the War Office, who will convert it into an orthopœdic centre. The hospital was liberally supported by every county in Wales, and it is understood that a meeting of the subscribers will shortly be held in order to decide what shall be done with the substantial balance in hand, which will be further augmented by the sum allowed by the War Office for the buildings.

### LEICESTER SHOWS THE WAY.

In the thought that before long a considerable activity in the building trade would be experienced, the governors of the Leicester Royal Infirmary have lost no time in inviting tenders for a new pathological department, with mortuary, post-mortem room, cold store, and viewing chapel, to be erected under the supervision of Messrs. Pick, Everard and Keay, architects. Out of a number of local firms selected to compete seven tendered, the price being accepted at £6,700. With one exception, the difference between the tenders was £400 only. The estimated cost in 1914 was approximately £4,000, so that the increase is 60 per cent. above pre-war figures. The information now given will be of interest to committees who have building schemes in comtemplation.

### SANATORIUM OR OPEN-AIR SCHOOL?

The Notts County Council contemplate providing two new buildings for the residential treatment of pulmonary tuberculosis, one of which is to be for advanced cases, and the other for children. The peculiar clinical characters of juvenile phthisis seem to make a children's sanatorium hardly an urgent measure. Roughly speaking, the moderate case of consumption in a child will get perfectly quiescent with merely removing it from school; while the severe one will often end fatally in spite of sanatorium treatment. In the best event, both will relapse with certainty when sent back to an ordinary school. Now in an open-air school, the little consumptive can get both health and learning, and an open-air school as a juvenile anti-tuberculosis agency is therefore worth far more than a sanatorium. If adults were as resistant as children to consumption, the whole tuberculosis problem would be immensely simplified.

### MINERS AND THE VOLUNTARY SYSTEM.

A gratifying response to the needs of the Beckett Hospital, Barnsley, resulted from a recent meeting at which the hospital's affairs were explained to the branch secretaries of the Yorkshire Miners' Association. Mr. A. Whitham, the energetic chairman of the hospital, frankly explained how the annual deficit had arisen, and it was all but unanimously decided to recommend a weekly contribution of one penny from the men, and one halfpenny from the boys, for the hospital's benefit. Mr. Whitham believed that if this plan were adopted the hospital's income would be sufficient to pay the increased cost of stores and drugs, and would further enable "an honest and fair wage" to be paid to the staff, especially the nurses. One of the miners' representatives declared that no movement appealed to the men more than the hospital movement, and this seems to be generally true, for only in 1916, the colliery proprietors in their turn combined to pay the adverse balance on that year. It was pointed out by Mr. Whitham that most of the cases received were surgical, the most expensive, perhaps which a hospital can treat. The promptness with which the position was understood and the readiness to help show that the relation of the men to their hospital is excellent and public-spirited.

